# Higher Risk of Cardiovascular Diseases in Rheumatoid Arthritis Patients Without Methotrexate Treatment

**DOI:** 10.3389/fphar.2021.703279

**Published:** 2021-11-05

**Authors:** Karel Hloch, Martin Doseděl, Jurjen Duintjer Tebbens, Lenka Žaloudková, Helena Medková, Jiří Vlček, Tomáš Soukup, Petr Pávek

**Affiliations:** ^1^ Department of Social & Clinical Pharmacy, Faculty of Pharmacy in Hradec Kralove, Charles University, Prague, Czechia; ^2^ Department of Biophysics & Physical Chemistry, Faculty of Pharmacy in Hradec Kralove, Charles University, Prague, Czechia; ^3^ Institute of Clinical Biochemistry and Diagnostics, University Hospital Hradec Kralove, Hradec Kralove, Czechia; ^4^ 2nd Department of Internal Medicine – Gastroenterology, Division Rheumatology, Faculty of Medicine & University Hospital in Hradec Kralove, Charles University, Hradec Kralove, Czechia; ^5^ Department of Pharmacology & Toxicology, Faculty of Pharmacy in Hradec Kralove, Charles University, Prague, Czechia

**Keywords:** rheumatoid arthritis, methotrexate, methotrexate discontinuation, cardiovascular diseases, cardiovascular risk factors

## Abstract

Cardiovascular diseases (CVDs) lead to higher morbidity and mortality in rheumatoid arthritis; thus, we aimed to determine whether patients who had discontinued methotrexate treatment before the study enrollment (group MTX 0) were at a higher risk of CVD than patients treated with methotrexate at the time of the data collection (group MTX 1). A retrospective, prospective, observational, cross-sectional study was conducted. A total of 125 patients were enrolled in the study. Patients from the MTX 0 group (*n* = 35) were not treated with methotrexate for 7.54 (SD ± 4.21) years in average. Medical documentation as well as information taken in patient examinations during regular rheumatologist visits was used to obtain the required data. The composite of any CVD occurred less frequently in patients in the MTX 1 group than in the MTX 0 group (18.8 vs. 40.0%, OR 0.35, 95% CI, 0.15 to 0.83; *p* = 0.017) with a non-significant trend after adjustment for other treatments, which differed between study groups at the baseline (*p* = 0.054). Significant difference was found for the reduction of myocardial infarction in the MTX 1 group compared to the MTX 0 group (3.5 vs. 14.3%, OR 0.22, 95% CI, 0.05 to 0.97; *p* = 0.046). There were 4 deaths (4.7%) in the MTX 1 group as compared with 7 (20.0%) in the MTX 0 group (OR 0.20, 95% CI, 0.05 to 0.73; *p* = 0.015). Our results demonstrate that patients who discontinued methotrexate treatment are at a significantly higher risk of CVD and all-cause mortality. Based on our findings, we recommend stricter control of CVD in cases of methotrexate discontinuation.

## Introduction

Rheumatoid arthritis (RA) is an autoimmune disease typically connected with chronic inflammation of the joints, causing their swelling and pain; RA leads to reduced mobility and increased mortality (Toledano et al., 2012). The prevalence of RA is about 1% in the general population with a correlation between women and men at the ratio of 3:1 (Soukup et al., 2015). Methotrexate (MTX), a drug with immunosuppressive activity and a member of the group of conventional synthetic disease–modifying antirheumatic drugs (csDMARDs), is the most frequent drug of choice used in RA therapy (Shinde et al., 2014).

General cardiovascular (CV) risk factors, such as dyslipidemia, obesity, diabetes mellitus (DM), hypertension, cigarette smoking, and physical inactivity, do not fully explain increased mortality due to cardiovascular diseases (CVDs) in RA patients (Choi et al., 2002; Schieir et al., 2017). A 2-fold higher relative risk of myocardial infarction (MI) has been found in female patients with RA compared to non-RA patients with the same level of general risk factors, and an even more 3-fold higher relative risk for women who have had RA for more than 10 years (Solomon et al., 2003). Thus, to obtain accurate results, general risk factors as well as markers of RA severity must be taken into consideration (Solomon et al., 2010). Indeed, absolute CV risk in patients with RA is higher in general and considered comparable with patients 5–10 years older without RA, or patients without RA suffering from DM (Peters et al., 2009).

A crucial role in higher morbidity and mortality in RA patients has been associated with increased inflammatory activity, with related CVD causing premature death in more than 50% of the cases (Symmons and Gabriel, 2011). Chronic inflammation leads to a more progressive development of atherosclerotic vascular disease measured as intima-media thickness (Ambrosino et al., 2015). Both innate and adaptive immunity are involved in pro-atherosclerosis activity and, together with the fact that RA is a disease with a well-proven ability to modify human immunity, this connection seems to be underlying for the development of CVD as well as for prevention and treatment options (Mankad, 2015). Therefore, RA has been considered an additional CV risk factor (Ku et al., 2009).

We hypothesized that RA patients without MTX treatment are at a higher risk of total CVD development, including angina pectoris (AP), MI, congestive heart failure (HF), arrhythmia, stroke, peripheral arterial disease (PAD), or sudden cardiac death, as well as individual CVDs. Moreover, we evaluated CV risk factors for the development of CVD (DM, hypertension, dyslipidemia, gout, hyperuricemia, and metabolic syndrome). To improve the understanding of the MTX treatment effect on CVD risk evaluation in RA patients, we examined three different CVD score lists from three sets of guidelines: Systematic Coronary Risk Evaluation (SCORE), the American College of Cardiology/the American Heart Association (ACC/AHA) CV risk score, and Reynolds Risk Score (RSC).

## Methods

### Study Design

A monocentric, regional, observational, retrospective, prospective, cross-sectional study was conducted.

### Settings

Patients were selected from 198 patients enrolled in our previous study (Soukup et al., 2015) (all RA patients available in the University Hospital at the beginning of the study fulfilled the ACR 1987 RA criteria and were currently treated or had been previously treated with MTX). All data were gathered from September, 01, 2016, to May, 31, 2017. Medical documentation was searched to obtain personal, family, and drug anamnesis. During a regular visit to a rheumatologist, each patient was examined, with basic characteristics and values connected with CV risks being recorded. Blood pressure (using a certificated and calibrated digital brachial tonometer), weight, height, and waist and hip circumference were measured. Fasting blood samples were collected and analyzed at the Institute of Clinical Biochemistry and Diagnostics, University Hospital Hradec Kralove. Analyses of total serum level cholesterol, LDL, HDL, triacyl glycerides, fasting glycemia, glycated hemoglobin, ions (Na^+^, K^+^, and Cl^−^), uric acid, creatinine, ESR, hsCRP, and RF were performed. Creatinine clearances were calculated according to the chronic kidney disease epidemiology collaboration (CKD-EPI) formula. Each patient’s medical records were searched for any kind of CVD and CVD risk factors. In addition, patients were questioned regarding daily physical activity as well as their smoking habits to obtain demographic data. All deceased patients (13) from the previous study were enrolled, and meaningful data from medical documentation was recorded together with the cause and date of the patient’s death. For these 13 patients, CV risk factors were not evaluated, as physical examination and laboratory data were not available.

### Participants

Patients from our previous study (Soukup et al., 2015), who visit rheumatologic outpatient clinics at the University Hospital of Hradec Králové, with the diagnosis of RA during the data collection, and who fulfill the inclusion and exclusion criteria were enrolled together with deceased participants from the above mentioned study. All these patients were selected because their MTX treatment statuses were known from the time of RA diagnosis. Patients met the inclusion criteria if they were currently or had been previously treated with MTX. There were no exclusion criteria being applied. All patients fulfilled the criteria put forth by the American College of Rheumatology (ACR) 1987 RA (Arnett et al., 1988).

In all the patients, CVD risk factors and CVD occurrences were recorded. Rheumatoid factors (RFs), antinuclear antibodies (ANAs), as well as antibodies against cyclic citrullinated peptide (ACCP) were evaluated. RA disease activity was measured at the time of study enrollment primarily using a disease activity score for 28 joints (DAS28) calculated using the erythrocyte sedimentation rate (ESR). For the determination of body-wide inflammation activity, ESR and high-sensitivity C-reactive protein (hsCRP) serum levels were evaluated.

Patients were sorted into two groups according to their RA treatment status. Patients treated with MTX at the time of data collection were designated as MTX users (MTX 1 group), and those who had not been treated with MTX for various reasons were selected as non-users (MTX 0 group). All patients from the MTX 0 group were treated with MTX in their history of diagnosis, and discontinuation of MTX treatment was based on EULAR recommendations. All patients were adults of Caucasian origin and living in the Czech Republic.

### Outcome Variables

We considered those patients as CVD positive who had history of AP, MI, congestive HF, stroke, PAD, sudden cardiac death, and arrhythmia (patients without sinus rhythm and/or with an atrioventricular block of at least 2nd degree) in their medical records. Moreover, an electrocardiogram of each patient was performed at the time of study enrollment and evaluated to obtain previously undiagnosed patients with CVDs. For the evaluation of positive CV risk factor history, data from medical records together with typical medication for each condition (i.e., statins, angiotensin-converting enzyme inhibitor (ACEi), metformin) were used. Overall, six CV risk factors were determined – DM, dyslipidemia, hypertension, hyperuricemia, gout, and metabolic syndrome (American Heart Association criteria; (Grundy et al., 2005)). In addition, three different CV scores were calculated. For each score, only patients who fulfilled specific criteria could be enrolled. First of all, the Systematic Coronary Risk Evaluation (SCORE; Conroy et al., 2003) for the prediction of 10-year risk of fatal CVD was calculated for patients between 35 and 65 years of age; the other requirements were a plasma cholesterol level of 2.5–12.0 mmol/l and systolic blood pressure of 90–190 mmHg. The SCORE was measured using the online calculator from the National Authorization Centre for Clinical Laboratories at the Czech Medical Society of Jan Evangelista Purkyně (NASKL, 2009). The patients eligible for ACC/AHA CV risk calculation for a 10-year risk of heart disease or stroke (calculated via online calculator) were between 40- and 79-year-old with a total cholesterol level of 3.37–8.29 mmol/l, HDL 0.52–2.59 mmol/l, systolic blood pressure 90–200 mmHg, and diastolic blood pressure 30–140 mmHg (Heart Risk Calculator, 2013). The last measurement, the calculation of the Reynolds Risk Score predicting a future heart attack, stroke, or other major heart diseases in the next 10 years was calculated for patients between 45 and 80 years of age, with total cholesterol 3.6–10.3 mmol/l, HDL 0.8–4.0 mmol/l, systolic blood pressure 90–200 mmHg, and CRP 0.03–20 mg/l (Reynolds Risk Score, 2013).

### Bias

To reduce the selection bias, we enrolled patients from the previous study (Soukup et al., 2015), which included all patients with RA diagnosis at the University Hospital of Hradec Králové. No additional exclusion criteria were applied. Out of 198 eligible patients, data from 120 (60.6%) were collected. We conducted an observational study using medical records as an important data source; thus, information bias due to missing or outdated data was identified. To handle this potential bias source, a pre-specified questionnaire was filled by the patients during their regular visit to a rheumatologist.

Atherosclerosis as an important factor for CVD occurrence is a multifactorial disease affected by many conditions. Subsequently, all these conditions may act as potential confounding factors in CVD development. To minimize the influence of the confounding factors on the results of our study, we analyzed the differences between study groups for uncontrollable (age, gender) and modifiable (DM, hypertension, dyslipidemia, smoking, alcohol consumption, physical activity, inflammatory activity) risk factors as well as patients’ medication with proven CVD effects (ACEi, beta blockers, statins, glucose lowering drugs, and antiplatelet and anticoagulant drugs). In addition, to control for the variables that are statistically and significantly correlated with MTX (see Table 1., i.e., beta blocker treatment, leflunomide treatment, and other bDMARDs), a propensity score matching approach (PSM) was used.

**TABLE 1:** Comparison of baseline characteristics between groups of RA patients treated (MTX 1 group) or non-treated (MTX 0 group) with MTX.

	Patients evaluated (MTX 1; MTX 0)	All groups	MTX 1	MTX 0	*p* value
Total number of patients, n (%)	120 (85; 35)	120 (100)	85 (70.8)	35 (29.2)	
Age (years), mean ± SD	109 (81; 28)	61.26 ± 11.77	61.56 ± 11.68	60.39 ± 12.19	0.654
Female gender, n (%)	120 (85; 35)	87 (72.5)	59 (69.4)	28 (80.0)	0.238
Smoking (current), n (%)	109 (81; 28)	25 (22.9)	21 (25.9)	4 (14.3)	0.207
Smoking (past), n (%)	109 (81; 28)	22 (20.2)	16 (20.0)	6 (21.4)	0.849
BMI (kg/m2), mean ± SD	108 (80; 28)	26.36 ± 5.69	26.04 ± 4.18	27.27 ± 8.73	0.477
Exercise (more than 2.5 h moderate physical activity per week)	104 (77; 27)	86 (82.7)	64 (75.3)	22 (62.9)	0.528
Rheumatoid factor positive, n (%)	88 (67; 21)	60 (68.2)	49 (73.1)	11 (52.4)	0.075
ACCP positive, n (%)	70 (51; 19)	58 (82.9)	45 (88.2)	13 (68.4)	0.074
ANA positive, n (%)	85 (66; 19)	37 (43.0)	29 (43.9)	8 (40.0)	0.802
DAS28 in randomization, mean ± SD	90 (70; 20)	2.42 ± 1.07	2.41 ± 1.04	2.42 ± 1.18	0.940
Remission or low disease activity^a^	90 (70; 20)	67 (74.4)	51 (72.9)	16 (80.0)	0.518
SBP (mmHg), mean ± SD	109 (81; 28)	145.99 ± 17.67	146.83 ± 16.72	143.57 ± 20.32	0.403
DBP (mmHg), mean ± SD	109 (81; 28)	86.11 ± 10.79	86.00 ± 9.61	86.43 ± 13.86	0.880
Heart rate (bpm), mean ± SD	105 (79; 26)	69.53 ± 10.59	70.05 ± 10.64	67.96 ± 10.50	0.386
Hypolipidemic treatment, n (%)	120 (85; 35)	41 (34.2)	30 (35.3)	11 (31.4)	0.654
Antihypertensive treatment, n (%)	120 (85; 35)	70 (58.3)	47 (55.3)	23 (65.7)	0.293
Antidiabetic treatment, n (%)	120 (85; 35)	13 (10.8)	6 (7.1)	7 (20.0)	0.053
ACEi/AT1 blockers treatment, n (%)	120 (85; 35)	54 (45.0)	41 (48.2)	13 (37.1)	0.266
Beta blockers treatment, n (%)	120 (85; 35)	42 (35.0)	23 (27.1)	19 (54.3)	0.004*
NSAID treatment, n (%)	120 (85; 35)	82 (68.3)	62 (72.9)	20 (57.1)	0.090
Corticoid treatment, n (%)	120 (85; 35)	85 (70.8)	59 (69.4)	26 (77.1)	0.393
Corticoid dose (mg), mean ± SD	120 (85; 35)	5.43 ± 3.06	5.15 ± 2.49	6.02 ± 4.01	0.308
Leflunomide treatment, n (%)	120 (85; 35)	18 (15.0)	6 (7.1)	12 (34.3)	0.0001*
Plaquenil treatment, n (%)	120 (85; 35)	16 (13.3)	9 (10.6)	7 (20.0)	0.168
Cyclosporine A treatment, n (%)	120 (85; 35)	4 (3.3)	2 (2.4)	2 (5.7)	0.579
Sulfasalazine treatment, n (%)	120 (85; 35)	4 (3.3)	3 (3.5)	1 (2.9)	0.999
bDMARD treatment, n (%)	120 (85; 35)	31 (25.8)	18 (21.2)	13 (37.1)	0.069
Anti–TNF-α bDMARDs, n (%)	120 (85; 35)	18 (15.0)	12 (14.1)	6 (17.1)	0.673
Other bDMARDs, n (%)	120 (85; 35)	13 (10.8)	6 (7.1)	7 (20.0)	0.038*

a According to EULAR criteria, remission or low disease activity was considered DAS28 < 3.2. n, number; SD, standard deviation; BMI, body mass index; ACCP, anti-cyclic citrullinated peptide; ANA, antinuclear antibody; DAS28, disease activity score 28; SBP, systolic blood pressure; DBP, diastolic blood pressure; ACEi, angiotensin-converting enzyme inhibitor; AT1, angiotensin receptor 1; bDMARDs, biologic disease–modifying anti-rheumatic Drugs; MTX 1, patients treated with MTX at the time of data collection; MTX 0, patients without MTX treatment at the time of data collection.

*Values are statistically significant (p < 0.05).

### Statistical Analysis

The differences between groups with the absence or presence of MTX treatment were evaluated using independent two-samples *t*-test for quantitative variables, and for qualitative variables we used the χ2-test or, when its assumptions were not satisfied, we used Fisher’s exact test. When it was necessary to assess, in addition, the effect size of MTX treatment’s absence/presence with respect to dichotomous outcome measures, the univariate logistic regression analysis was used, and results were expressed in terms of an odds ratio (OR) with a 95% confidence interval (CI). To control for the variables that were statistically significant in Table 1 using a propensity score-matching approach, every patient with MTX was matched with the patient without MTX whose propensity score was closest. To assess differences between mortality in the absence or presence of MTX treatment, survival analysis using a log-rank test was performed. Statistical significance was considered at *p*-value equal to 0.05. All statistical analyses were carried out using the SPSS statistical package, version 16 (SPSS Inc., Chicago, Illinois, United States).

## Results

**FIGURE 1:**
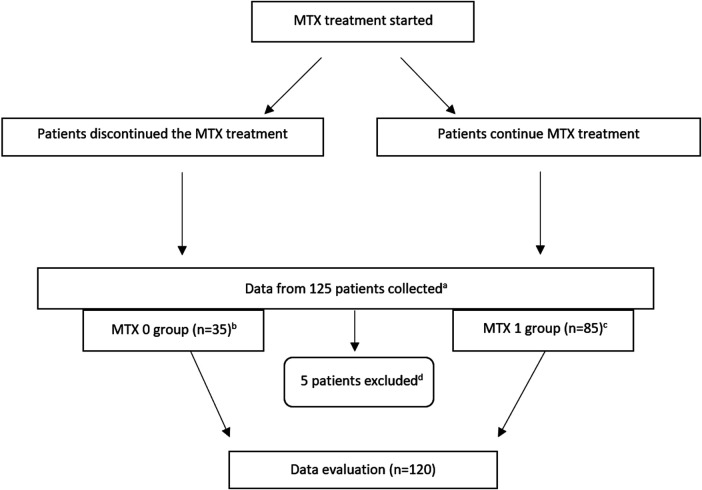
Flow diagram. **(A)** Patients from previous study (Soukup et al., 2015) who visited rheumatologic outpatient clinics at the University Hospital of Hradec Králové with the diagnosis of RA during the data collection and who were treated with MTX at the time of data collection or anytime in the past, and all deceased participants from the abovementioned study. **(B)** Average time from MTX discontinuation in the MTX 0 group was 7.54 (SD ± 4.21) years. **(C)** In the MTX 1 group, the patients had been treated with MTX for an average of 10.9 years (SD ± 5.0). **(D)** Two patients refused to be enrolled in the study, one patient could not be examined due to hospitalization for active oncologic disease at the time of data collection when medical records from two deceased patients were not available. n, number; MTX, methotrexate; MTX 1, patients treated with MTX at the time of data collection; MTX 0, patients without MTX treatment at the time of data collection.

A total of 125 patients were enrolled in the study, with 120 patients eligible for the statistical analysis (Figure 1). Two patients refused to be enrolled in the study, and one patient could not be examined due to hospitalization for active oncological disease at the time when data collection and medical records from 2 deceased patients were not available. 35 patients were assigned to the MTX 0 group and 85 were assigned to the MTX 1 group. The average time from MTX discontinuation in the MTX 0 group was 7.54 (SD ± 4.21) years. The minimum and maximum period from MTX discontinuation was 1 and 20 years, respectively. The reason for MTX discontinuation was known for 25 patients with side effects as the leading cause in 72% cases (side effect *n* = 18, inefficacy *n* = 3, loss of efficacy *n* = 2, others = 2). In the MTX 1 group, the patients had been treated with MTX for an average of 10.9 years (SD ± 5.0). The minimum and maximum period of MTX treatment was 4 and 27 years, respectively. In total, 13 patients were deceased at the time of data collection, 7 from the MTX 0 group (20.0%) and 4 from the MTX 1 group (4.6%); and for the last 2 patients, no medical records were available. The CV cause of death was reported in 7 patients (5 from the MTX 0 group and 2 from the MTX 1 group).

The patients’ baseline characteristics are presented in Table 1. In a comparison of the two study groups, no significant difference was found in most of the parameters. The patients in the MTX 0 group were more frequently treated with betablockers (54.3 versus 27.1%, *p* = 0.004), leflunomide (34.3 versus 7.1%, *p* = 0,0001), and with non–anti–TNF- α bDMARDs (17.1 versus 14.1%, *p* = 0.038). The laboratory parameters are summarized in Table 2, with no significant differences found between the groups. Importantly, there was no significant difference between values related to RA activity such as hsCRP (*p* = 0.120) and ESR (*p* = 0.095) for the MTX 0 group versus the MTX 1 group.

**TABLE 2:** Comparison of laboratory test results between groups of RA patients treated (MTX 1 group) or non-treated (MTX 0 group) with MTX.

	Patients evaluated (MTX 1; MTX 0)	All groups	MTX 1	MTX 0	*p* value
Cholesterol (mmol/l), mean ± SD	104 (78; 26)	5.29 ± 1.13	5.24 ± 1.10	5.45 ± 1.22	0.412
LDL (mmol/l), mean ± SD	100 (75; 25)	3.15 ± 0.98	3.17 ± 0.94	3.11 ± 1.12	0.823
HDL (mmol/l), mean ± SD	101 (76; 25)	1.77 ± 0.52	1.73 ± 0.51	1.87 ± 0.57	0.277
TAG (mmol/l), mean ± SD	101 (76; 25)	1.60 ± 0.93	1.53 ± 0.88	1.81 ± 1.06	0.198
hsCRP (mg/l), mean ± SD	105 (79; 26)	5.75 ± 9.50	4.41 ± 4.60	9.82 ± 16.93	0.120
ESR (mm/h), mean ± SD	98 (75; 23)	14.32 ± 9.87	13.12 ± 8.19	18.26 ± 13.53	0.095
Creatinine (μmol/l), mean ± SD	105 (79; 26)	81.56 ± 30.32	80.13 ± 21.20	85.88 ± 47.77	0.557
Glycemia (mmol/l), mean ± SD	104 (78; 26)	5.54 ± 1.70	5.31 ± 0.90	6.23 ± 2.90	0.123
HbA1c (mmol/l), mean ± SD	96 (73; 23)	37.49 ± 7.78	36.73 ± 6.26	39.91 ± 10.68	0.185
GFL (ml/s/1.73 m^2^), mean ± SD	104 (78; 26)	1.30 ± 0.31	1.30 ± 0.29	1.29 ± 0.40	0.881
Uric acid (μmol/l), mean ± SD	104 (78; 26)	264.66 ± 103.45	259.24 ± 112.99	280.92 ± 75.80	0.364

n, number; SD, standard deviation; LDL, low-density lipoprotein; HDL, high-density lipoprotein; TAG, triacylglycerol; hsCRP, high-sensitivity C-reactive protein; ESR, erythrocyte sedimentation rate; HbA1c, glycated hemoglobin; eGFR, estimated glomerular filtration rate; MTX 1, patients treated with MTX at the time of data collection; MTX 0, patients without MTX treatment at the time of data collection.

**FIGURE 2:**
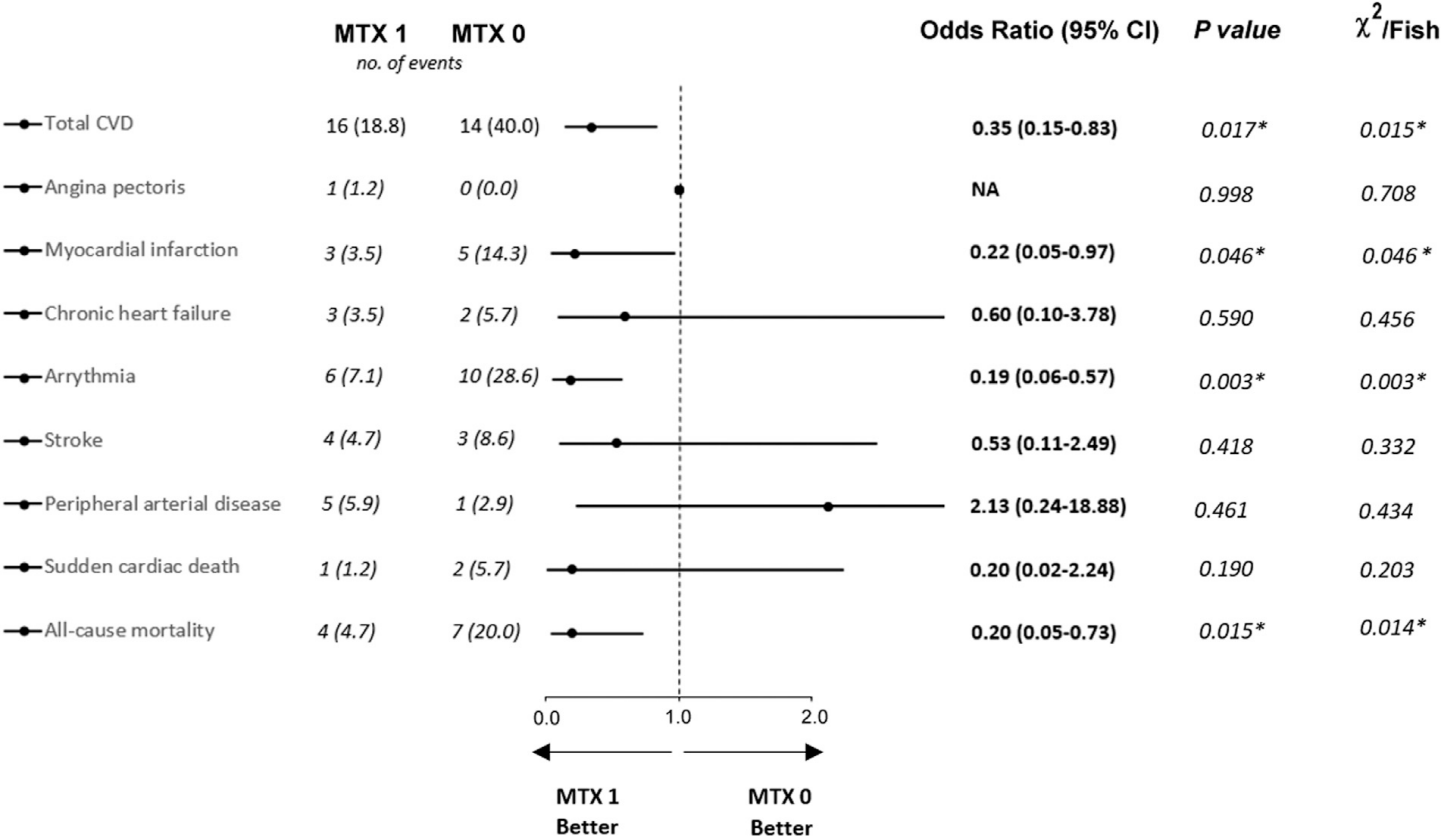
Proportion of cardiovascular diseases and all-cause mortality between groups of RA patients treated (MTX 1 group) or non-treated (MTX 0 group) with MTX. * Values are statistically significant (*p* < 0.05). Data are available from all 120 patients (MTX 1 *n* = 85; MTX 0 *n* = 35). NA, not available; CVD, cardiovascular disease; MTX 1, patients treated with MTX at the time of data collection; MTX 0, patients without MTX treatment at the time of data collection.

A composite of CV outcome events consisted of AP, MI, congestive HF, arrhythmia, stroke, PAD, or sudden cardiac death which occurred in 16 (18.8%) patients who were assigned to the MTX 1 group and 14 (40%) patients who were assigned to the MTX 0 group (Figure 2). For the comparison of these two groups, the odds ratio (OR) for the primary outcome was 0.35, (95% confidence interval [CI], 0.15–0.83; *p* = 0.017).

Individual CVD events occurred in fewer patients in the MTX 1 group in cases of MI (3.5 versus 14.3%; OR 0.22, 95% CI, 0.05–0.97; *p* = 0.046) and arrhythmia (7.1 versus 28.6%; OR 0.19, 95% CI, 0.06–0.57; *p* = 0.003). The other CVD events did not show a significant difference between the two groups. The individual CVD events are described in detail in Figure 2.

**FIGURE 3:**
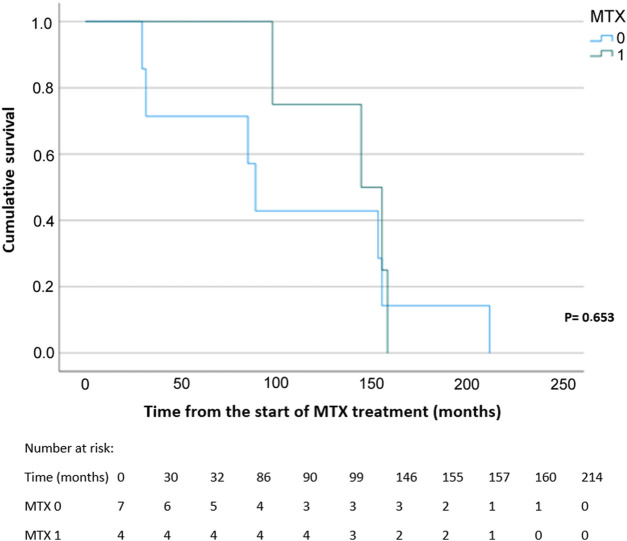
Kaplan–Meier curve and survival analysis for all-cause mortality probability using the log-rank test. MTX, methotrexate; MTX 1, patients treated with MTX at the time of data collection; MTX 0, patients without MTX treatment at the time of data collection.

Statistically significant difference was found for all-cause mortality. There were 4 deaths (4.7%) in the MTX 1 group as compared with 7 (20.0%) in the MTX 0 group (OR 0.20, 95% CI, 0.05–0.73; *p* = 0.015). However, the Kaplan–Meier analysis showed that there were no statistically significant differences in survival probability between the MTX 0 and MTX 1 groups (Figure 3).

**FIGURE 4:**
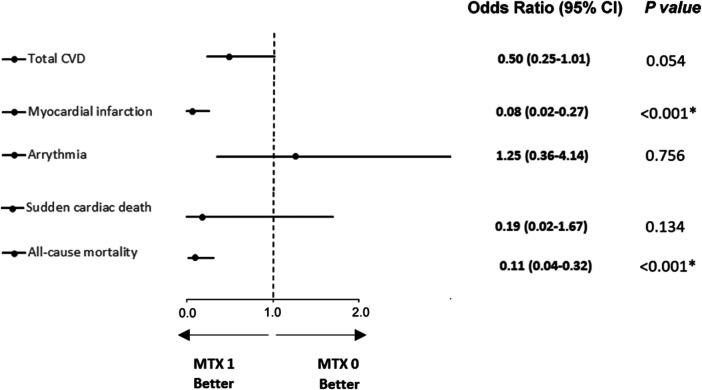
Logistic regression analysis after propensity score matching for CVDs and all-cause mortality between groups of RA patients treated (MTX 1 group) or non-treated (MTX 0 group) with adjustment for leflunomide, beta blockers, and other bDMARD treatments. * Values are statistically significant (*p* < 0.05). CVD, cardiovascular disease; MTX 1, patients treated with MTX at the time of data collection; MTX 0, patients without MTX treatment at the time of data collection.

After adjustment for statistically significant differences observed in Table 1 using PSM, a non-significant trend was found in total CVD (OR 0.50, 95% CI, 0.25–1.01; *p* = 0.054) for the MTX 1 group. Moreover, PSM analysis demonstrates statistically significant results for MI (OR 0.08, 95% CI, 0.02–0.27) and all-cause mortality (OR 0.11, 95% CI, 0.04–0.32). Results for sudden cardiac death (OR 0.19, 95% CI, 0.02–1.67) and arrhythmia (OR 1.25, 95% CI, 0.36–4.14) did not reach statistical significance between the study groups (Figure 4). Due to the limited number of cases, PSM was not conducted for AP, HF, CMP, and PAD.

### CV Risk Factors for the Development of CVD and CVD Score Lists

Outcomes defined as risk factors and risk factor scores for the development of CVD are summarized in Table 3.

**TABLE 3:** Risk factors for the development of cardiovascular diseases between groups of RA patients treated (MTX 1 group) or non-treated (MTX 0 group) with MTX.

	Patients evaluated (MTX 1; MTX 0)	All groups	MTX 1	MTX 0	*p* value
Diabetes mellitus I or II	109 (81; 28)	11 (10.1)	5 (6.2)	6 (21.4)	0.011*
Dyslipidemia	109 (81; 28)	49 (45.0)	35 (43.2)	14 (50.0)	0.534
Arterial hypertension	109 (81; 28)	87 (79.8)	66 (81.5)	21 (75.0)	0.461
Hyperuricemia	109 (81; 28)	22 (20.2)	15 (18.5)	7 (25.0)	0.461
Gout	109 (81; 28)	2 (1.8)	2 (2.5)	0 (0)	0.401
Metabolic syndrome	100 (74; 26)	59 (59.0)	44 (59.5)	15 (57.7)	0.875
SCORE, mean ± SD	61 (46; 15)	3.10 ± 4.92	3.38 ± 5.25	2.30 ± 2.46	0.433
ACC/AHA CV risk score, mean ± SD	81 (64; 17)	14.07 ± 12.48	13.48 ± 11.34	16.54 ± 16.42	0.364
Reynolds risk score, mean ± SD	80 (61; 19)	9.35 ± 8.29	9.97 ± 9.41	7.45 ± 7.85	0.284

*Values are statistically significant (*p* < 0.05). SD, standard deviation; CV, cardiovascular; SCORE, Systematic Coronary Risk Evaluation; ACC/AHA, American College of Cardiology/American Heart Association; MTX 1, patients treated with MTX at the time of data collection; MTX 0, patients without MTX treatment at the time of data collection.

Statistically significant differences between the study groups in CVD risk factors were found only for both types of DM (21.4 versus 6.2%, *p* = 0.011) with higher incidence in the MTX 0 group.

The evaluation of the SCORE list, ACC/AHA cardiovascular risk score, and the Reynolds Risk Score did not show any significant differences between study groups (*p* = 0.433, 0.364 and 0.284, respectively).

## Discussion

The fact that CVD is involved in higher mortality in RA is well documented. A meta-analysis containing 24 studies showed increased mortality caused by CVD in about 50% of patients with RA as compared to the general population (Aviña-Zubieta et al., 2008). However, there is a lack of systematic evaluation for the impact of individual CVD in terms of methotrexate (MTX) treatment.

In total, 25% patients of our study cohort showed CVD in statistically significant lower proportion in patients treated with MTX in comparison with patients who had discontinued MTX treatment before the study enrollment (*p* = 0.017). 40.0% of patients from the MTX non-treatment group and 18.8% from those treated with MTX developed a type of CVD, that is, defined as AP, MI, congestive HF, arrhythmia, stroke, PAD, or sudden cardiac death, with statistically significant reduction in crude analysis (*p* = 0.015; Figure 2) and a slightly non-significant trend after adjusted analysis (*p* = 0.054; Figure 3). These findings correlate with those of previous studies (Westlake et al., 2010; Micha et al., 2011; De Vecchis et al., 2016). Interestingly in the CIRT study, low-dose MTX compared to the placebo did not result in fewer composite endpoints consisting of major CV events (MACE) extended by hospitalization for unstable angina that led to urgent revascularization. This double-blind trial randomized very high-risk patients for CVD (a history of MI or multivessel coronary disease and DM type II or metabolic syndrome) without chronic inflammatory conditions such as RA. Moreover, MTX did not reduce levels of IL-1β, IL-6, and CRP. The discrepancy between our data for low-dose MTX in RA patients with data published in the CIRT study may suggest p heterogeneity of the inflammatory pathways (Ridker et al., 2019).

When we evaluated each single CVD, a statistically significant reduction was shown for MI for both crude and adjusted analysis (*p* = 0.046 and *p* < 0.001, respectively), and for arrhythmia in crude analysis with a proportion of ten and six events for the MTX non-treatment group and for those treated with MTX, respectively (*p* = 0.003) (Figure 2). This statistically significant result was not confirmed using PSM analysis (*p* = 0.356) (Figure 3). Larger studies confirmed a higher prevalence of arrhythmia, mostly atrial fibrillation, in patients with RA. The connection between arrhythmia and RA seems to be linked via different kinds of mechanisms, but the main cause is attributed to chronic inflammatory condition, which indirectly influences arrhythmia by a higher occurrence of CAD and HF, as well as via a direct pathway to cardiac electrophysiology (7,8).

A comparison of the risk factors for the development of CVD revealed a statistically significant difference between the study groups only in the case of both types of DM at the distribution of seven (25.0%) in the MTX non-treatment group and four (4.9%) in patients treated with MTX (*p* = 0.011). Although this comparison between the study groups showed a significant difference for DM 1st or 2nd type, this should not be a leading cause of higher CVD in patients without MTX treatment because of the comparable glycated hemoglobin blood level, which demonstrated sufficient DM treatment in both the studied groups (Table 2). Moreover, none of the diabetic patients were treated with SGLT-2 inhibitors or GLP-1 agonists, which could influence our results because of their proven effects on CVD.

Unlike the result for DM, other CV risk factors did not differ significantly between the groups (Table 3). These results are in accordance with previous literature findings, suggesting that the current treatment of risk factors for the development of CVD for patients with RA is not sufficient, as well as that RA disease activity plays a crucial role in the development of CVD (van Breukelen-van der Stoep et al., 2013).

Moreover, no significant differences were found between the study groups when the three different CVD score lists (SCORE, ACC/AHA CV risk score, and the Reynolds Risk Score) were compared (Table 3), which is in accordance with a previous article by Crowson et al. regarding the usefulness of risk scores to estimate the risk of CVD in patients with RA (Crowson et al., 2012). Although the Reynolds Risk Score, which is calculated with the marker of inflammatory activity (hsCRP), was numerically higher in patients treated with MTX, this fact does not demonstrate the superiority of this score list. Therefore, the hsCRP blood level does not seem to be sufficient for the prediction of the development of CVD; thus, RA disease activity should be considered as well to obtain more accurate results (del Rincon et al., 2001; Solomon et al., 2003; Gonzalez et al., 2008).

In our sample, patients untreated with MTX had insignificantly higher body-wide inflammation as measured using hsCRP and ESR, a result which could be connected with increased CVD and CV risk factors (Wallberg–Jonsson et al., 1999; Ridker et al., 2000). However, the activity of the disease calculated using the DAS28 scale was similar in both study groups, suggesting no difference in RA activity between them. Moreover, the same proportion of patients across the study groups showed RA remission (58.6 versus 60.0%) according to the EULAR classification (Table 2) (Smolen et al., 2017).

In our study, out of the entire study population, 35 patients (29.2%) were not treated with MTX. This proportion correlates with the previous published finding that MTX failure occurs in approximately one quarter of patients within 12 months (Bluett et al., 2018). This induces a high risk of CVD in patients due to insufficient inflammatory control, especially from the onset of the disease to the determination of an adequate RA treatment for these patients. This assumption is in accordance with previous data indicating that patients for whom MTX failed as a first-line strategy have significantly more radiographic joint damage (van der Kooij et al., 2007).

The second most common csDMARD used in our study was leflunomide, which was used in a significantly higher proportion in patients who were not treated with MTX (Table 1). Leflunomide does not seem to have a potential for increasing MI as a one of the leading causes of increased total CVD in our sample (Suissa et al., 2006). On the other hand, leflunomide showed a higher proportion of intima-media thickness and prevalence of plaques compared to MTX ≥ 20 mg/wk, biologics, or CsA (Kisiel et al., 2015). For the most frequent CVD and arrhythmias, no relevant data confirming the association with leflunomide use were found. In addition, glucocorticoids with their strong anti-inflammatory activity may have potentially influenced the results of our study, as side effects include hypertension and dyslipidemia; however, the CV risk from these substances is doubtful because of their anti-RA disease activity (Nurmohamed et al., 2002). Moreover, the number of patients treated with glucocorticoids did not differ between the study groups nor did the total glucocorticoid dosage used (Table 1).

Biological treatment is used once when standard therapy fails to achieve remission or at least lower disease activity. Chief among the effects of bDMARDs is the suppression of inflammation activity via the inhibition of TNF-α or other pro-inflammatory cytokines (Atzeni et al., 2013). Current evidence suggests that pro-inflammatory cytokines are involved in the development and rupturing of atherosclerotic plaques (Crowson et al., 2013).

The proportion of bDMARD treatment between our study groups did not reach a statistically significant difference. However, the number of patients treated with bDMARDs in our sample is not negligible (25.8%) (Table 1). This number is not sufficient to calculate the effect of this treatment on CVD, although a trend indication was found for biological treatment with only 12.9% patients who manifested any CVD in comparison with 30.4% CVD episodes in patients not treated by bDMARDs. As biological treatment, particularly, TNF-i seems to have a high impact on the reduction of CVD development (Khraishi et al., 2013; Humphreys et al., 2016), and the early start of bDMARD treatment may be beneficial. Moreover, the combination of bDMARDs with csDMARDs also demonstrated a better CVD prognosis compared with monotherapy (Singh et al., 2017; Xie et al., 2018).

Biological treatments as well as other csDMARDs have a strong anti-inflammatory activity alone or in combination; however, our results suggest that patients treated with MTX benefit more from therapy. Subclinical atherosclerosis, which develops before the diagnosis of RA, together with longer time needed to achieve remission or at least lower disease activity, seems to play an important role in CV morbidity (Maradit-Kremers et al., 2005; Smolen et al., 2017; Mackey et al., 2018). Moreover, the lower activity of RA disease, which is achieved earlier in MTX responders, improves endothelial dysfunction (de Groot et al., 2015). Through its vasodilatation effect, MTX also interferes with the adenosine mechanism, which plays an important role in cardiac and vascular biology. In addition, adenosine also affects heart contractility via the decrease of spontaneous depolarization in the sinus node. In summary, a higher level of adenosine in MTX treatment leads to the upregulation of various cholesterol efflux transporter proteins. Consequently, the transport of cholesterol from the periphery to the liver and the decreased formation of foam cells relates to a decrease in the risk of CVD (Reiss et al., 2019).

The findings of this study should be seen in the light of certain limitations. First, the study has limited number of patients coming from one geographical region, and all of them were treated at the same hospital. Although patients were enrolled and split into groups of 7 physicians, potential influence of the regional nature on the study cannot be ruled out. Second, some patients’ characteristics were missing, as the data were obtained from their medical records which did not contain complete data in some of the cases. To minimalize the number of such missing values, we asked the patients to fill in a questionnaire (e.g., current pharmacotherapy) during their regular visit to a rheumatologist. Third, due to the retrospective data of our study, some factors may have changed and affected the primary outcomes, such as continuous use of CVD preventing medications, RA disease activity, and exercise. Moreover, based on the cross-sectional design of the study, we do not know when the episode of CV event occurred. Thus, CV events in the MTX 0 group could have occurred at the time of a previous MTX use or in the consecutive period after MTX discontinuation. To eliminate this factor, a future prospective study is needed. Fourth, residual confounding can account for some of the observed associations. We used PSM for variables with a statistically significant difference in baseline characteristics (Table 1) to minimize this bias.

## Conclusion

According to recent recommendations, controlling risk factors for CV morbidity and managing the activity of RA for long-term and sustained remission are the two methods to reduce CV morbidity and mortality. Data from our study show that there is a higher risk of CVD and all-cause mortality in patients who discontinued MTX treatment in comparison with patients continuously treated with MTX. No such relationship was found for general CV risk factors or different CV risk scoring scales. In conclusion, our data support current EULAR recommendations for using MTX in the first-line treatment strategy. On the other hand, in clinical practice, a part of the patients with RA are not treated using MTX. First, these patients have contraindications of MTX. Second, patients developed adverse events. Third, MTX was withdrawn due to inefficacy. In case of MTX inefficacy, it is possible to continue with a combination of MTX and other DMARDs. According to the results of our study, we recommend stricter control of CVD to justify cases of MTX discontinuation. Nevertheless, due to the limited number of participants, high-quality randomized clinical trials are required to verify the effect of MTX in CVD prevention.

## Data Availability

The raw data supporting the conclusions of this article will be made available by the authors, without undue reservation.
